# Hope for HIV control in southern Africa: The continued quest for a vaccine

**DOI:** 10.1371/journal.pmed.1002241

**Published:** 2017-02-28

**Authors:** Linda-Gail Bekker, Glenda E. Gray

**Affiliations:** 1 Desmond Tutu HIV Centre, University of Cape Town, Cape Town, South Africa; 2 South African Medical Research Council, Cape Town, South Africa

## Abstract

In a Perspective, Linda-Gail Bekker and Glenda Gray discuss HIV vaccine development.

It is estimated that almost 2 million new HIV infections occurred in the world in 2015 [1]. Southern and eastern Africa, with 6.2% of the world’s population, bear a disparate half of the world’s HIV infection burden and would benefit greatly from inexpensive innovations aimed at curtailing the epidemic. A recent modelling study showed that introducing a partially (30%) effective vaccine for HIV in resource-limited settings such as southern Africa would result in an estimated 67% reduction in HIV incidence compared to a non-vaccine scenario [2]. As sub-Saharan Africa has the highest incidence of HIV infection in the world, that the introduction of a vaccine with only partial efficacy could have such a dramatic effect, despite the existing availability of comprehensive prevention methods, is strongly persuasive for the pursuit of a vaccine-based approach [3].

Whilst there is great optimism that increasing access to antiretroviral treatment in the region will reduce infection incidence, there is also recognition that epidemic control will not be achieved without a substantial and sustained scale-up of additional primary prevention resources [1]. There are challenges to HIV prevention in resource-limited settings that a vaccine alone is well positioned to meet. These include the rate of HIV infections and the scale and complexity of the HIV epidemic in the region, juxtaposed with ailing health systems ill equipped to respond effectively. Challenges with antiretroviral drug therapy adherence, poor linkage to care following diagnosis, multiple and diverse vulnerable populations who require population-specific services (such as women, adolescents, and men who have sex with men [MSM]), stigma, and discrimination, as well as generally limited health care facilities and human capital, impair the region’s capacity to manage the scale of the epidemic.

Even with the success of pre-exposure prophylaxis (PrEP) demonstration projects and the encouraging results emerging, the extent of protection relies on fidelity to adherence, continuous uninterrupted access, and sustainable resources for provision [4]. It is well documented that in resource-restricted areas, where education levels and access to health care are low, reliance on behavioural and structural support is also an enormous challenge. A vaccine, even if partially effective, is a way of filling these prevention gaps in a cost-effective manner. Whilst countries in this region must find ways to access all the available opportunities that the modern HIV prevention toolkit has on offer, such a vaccine could significantly change the prevention landscape.

## The RV144 HIV vaccine trial and links to further trials

Importantly, a partially efficacious vaccine such as the one described in the recent modelling study has already been demonstrated. The RV144 vaccine trial was conducted amongst 16,395 heterosexual HIV-uninfected Thai adults using an ALVAC-HIV and AIDSVAX B/E gp120 boost regimen, and RV144 was the first vaccine to show any efficacy in reducing HIV acquisition, with a 60.5% (95% CI 22–80) efficacy within 12 months and a 31.2% (95% CI 1.1–52.1) efficacy after 3.5 years [5].

Thailand is dominated by an HIV clade B/E epidemic, and as the RV144 vaccine was designed to meet the criterion of protection against this HIV clade, it was vital to consider whether this vaccine regimen would bring about equivalent results in other clades. In particular, clade C is of interest as just under half of HIV-infected individuals possess this subtype. The HVTN 097 study replicated the RV144 vaccine regimen in South Africa, a clade C-dominated region, and compared cellular and humoral responses to age- and sex- matched RV144 Thai participants. The investigators found that, despite large differences between participant population ethnicity, HIV clade, and predominant mode of transmission, the response rates were equivalent if not greater than those induced in the Thai study [6]. A parallel study (HVTN 100) developed a clade C ALVAC-HIV and bivalent subtype C gp120/MF59 vaccine for specific use in clade C-dominant regions, and conducted a similar phase 1–2 preventative vaccine trial in low-risk South African adults [7]. Preliminary results suggest a strong vaccine-induced immune response, greater than that seen in the RV144 regimen, giving the green light to advance further development of this vaccine regimen in a pivotal phase IIb/III clinical trial (HVTN 702), which commenced in November 2016 in South Africa. HVTN 702 will evaluate the vaccine’s efficacy, tolerability, and safety in 5,400 HIV-uninfected adults over 24 months [8].

## Broadly neutralizing antibodies

The breakthrough with RV144 was unexpected because, in the preclinical studies, ALVAC-HIV showed unimpressive immunogenicity and the protein boosts alone were unable to prevent HIV acquisition [9,10]. Additionally, there was a notable absence of stimulation of broadly neutralizing antibodies (BNAbs), the current popular target of new HIV vaccine research [11]. BNAbs are a favoured topic in the HIV vaccine field because, once elicited, they are able to neutralize most strains of a viral pathogen, which would be highly desirable in the case of an HIV vaccine. BNAb responses do occur naturally in a small number of HIV-infected individuals, albeit years after infection. Key knowledge gaps have prevented a BNAb-focused vaccine approach, including the issues of how BNAbs develop and the mechanism by which the HIV virus drives their production. Isolated human samples from HIV-infected patients who do develop BNAbs have been studied, and this has provided some insight, from which immunogens have been designed [11]. A proof-of-concept clinical trial to test whether one particular BNAb known as VRC01, which binds to the CD4 binding region of the HIV envelope, is effective in preventing HIV infection in women in the southern and eastern African region is currently underway. A companion trial evaluating this concept is being carried out in the Americas in MSM and transgender women. These trials will define whether HIV transmission at the mucosal level can be averted [12].

## Moving from efficacy to implementation

The findings of the HVTN 702 trial have the potential to be a great leap in HIV prevention research, with vaccine efficacy results anticipated in early 2021. But, even if efficacious, a paramount challenge will be getting the vaccine out of the laboratory and to those people who need it most—particularly, difficult-to-reach populations in low- and middle-income countries. Notably, the cost of vaccine manufacturing will play a large role, as developing countries are already having to make tough decisions regarding optimal funding of HIV prevention and treatment programs. If a vaccine is at best only partially efficacious, the correct combination of preventative measures will need to be explored, likely on a population-specific basis, and choices made. From current predictions, however, a safe vaccine of even modest efficacy could be the game changer necessary to close the HIV prevention gap.

## Abbreviations

BNAb: broadly neutralizing antibody
MSM: men who have sex with men
PrEP: pre-exposure prophylaxis
